# A unique protein profile of peripheral neutrophils from COPD patients does not reflect cytokine-induced protein profiles of neutrophils *in vitro*

**DOI:** 10.1186/1471-2466-11-44

**Published:** 2011-09-06

**Authors:** Jeroen D Langereis, René C Schweizer, Jan-Willem J Lammers, Leo Koenderman, Laurien H Ulfman

**Affiliations:** 1 Department of Respiratory Medicine, University Medical Center, Heidelberglaan 100, 3584 CX Utrecht, the Netherlands

## Abstract

**Background:**

Inflammation, both local and systemic, is a hallmark of chronic obstructive pulmonary disease (COPD). Inflammatory mediators such as TNFα and GM-CSF are secreted by lung epithelium, alveolar macrophages and other inflammatory cells and are thought to be important contributors in the pathogenesis of COPD. Indeed, neutrophils are activated by these cytokines and these cells are one of the major inflammatory cell types recruited to the pulmonary compartment of COPD patients. Furthermore, these inflammatory mediators are found in the peripheral blood of COPD patients and, therefore, we hypothesized that TNFα/GM-CSF-induced protein profiles can be found in peripheral neutrophils of COPD patients.

**Methods:**

Using fluorescence 2-dimensional difference gel electrophoresis we investigated differentially regulated proteins in peripheral neutrophils from COPD patients and healthy age-matched control subjects. Furthermore, protein profiles from COPD patients were compared with those of neutrophils of healthy age-matched controls that were stimulated with TNFα and/or GM-CSF *in vitro*. Protein gels were compared using DeCyder 7.0 software.

**Results:**

We identified 7 significantly regulated protein spots between peripheral neutrophils from COPD patients and age-matched healthy control subjects. Stimulation of peripheral neutrophils with TNFα, GM-CSF or TNFα + GM-CSF *in vitro *resulted in 13, 20 and 22 regulated protein spots, respectively. However, these cytokine-induced protein differences did not correspond with the protein differences found in neutrophils from COPD patients.

**Conclusion:**

These results show that neutrophils from COPD patients have a unique protein profile compared to neutrophils from healthy age-matched controls. Furthermore, the neutrophil profiles of COPD patients do not reflect putative dominant signals induced by TNFα, GM-CSF or their combination. Our results indicate that systemic neutrophil responses in COPD patients are caused by a unique but subtle interplay between multiple inflammatory signals.

## Background

COPD is classified by the guidelines of the Global Initiative for Chronic Obstructive Lung Disease, which is based on lung function parameters: forced expiratory volume in 1 second (FEV_1_) and forced vital capacity (FVC) [1]. However, it has become increasingly clear that the GOLD classification does not represent the complex local and systemic inflammation in COPD [2-4]. Part of this inflammatory process is the secretion of inflammatory mediators by lung epithelium, alveolar macrophages and other inflammatory cells [5]. These inflammatory mediators affect the local tissue and attract inflammatory cells to the site of inflammation. For instance, alveolar macrophages secrete tumor necrosis factor-α (TNFα) [6] and granulocyte macrophage-colony stimulating factor (GM-CSF) [7] upon stimulation with cigarette smoke and increased levels of these cytokines are measured in the bronchiolar alveolar lavage (BAL) fluid, sputum or peripheral blood of COPD patients [8-12]. A central role for GM-CSF in smoke-induced inflammation was shown by intranasal administration of anti-GM-CSF antibodies to mice exposed to cigarette smoke, which reduced BAL fluid macrophages, neutrophils and TNFα synthesis [13]. Increased levels of TNFα were found in exhaled breath condensates [14], sputum [15] and serum of COPD patients [16-18].

Elevated cytokine levels in serum are frequently found in COPD patients [11,16-20]. However, these differences are often small compared to healthy controls, and the biological activity of these cytokines is dependent on the ratio with their naturally occurring inhibitors and other cytokines. Therefore, the use of peripheral neutrophils that have integrated all pro- and anti-inflammatory signals *in vivo *might be a more biologically relevant read-out to measure the systemic inflammatory status of a COPD patient. Previous studies in our laboratory showed that stimulation of neutrophils with either TNFα or GM-CSF *in vitro *resulted in differential expression of genes encoding chemokines and cytokines [21]. Various genes were similarly induced upon stimulation with TNFα or GM-CSF. More interestingly, the combination of these cytokines induced a unique mRNA pattern, which was distinct from the profiles induced by either cytokine alone. For instance, GM-CSF did not affect the expression of CD83 mRNA in control cells, but inhibited its expression induced by TNFα. This GM-CSF-induced inhibition was dose-dependent and was confirmed at the protein level by Western blot analysis [22]. These results show that integration of multiple cytokine signals can result in a distinct phenotype of the neutrophils.

Our study was designed to define the protein profiles of neutrophils found in COPD patients and to compare these with protein profiles found after *in vitro *stimulation. We performed fluorescence 2-dimensional (2D) difference gel electrophoresis (DIGE) on peripheral neutrophils from COPD patients and age-matched healthy controls and compared the differentially regulated proteins with differentially regulated proteins induced by TNFα and/or GM-CSF *in vitro*. We report 7 protein differences in neutrophils from COPD patients compared to neutrophils from healthy age-matched control subjects. TNFα, GM-CSF or TNFα + GM-CSF stimulation *in vitro *resulted in 13, 20 and 22 protein differences, respectively. Although cytokine stimulation of peripheral neutrophils *in vitro *showed differential protein expression, this did not correspond to differential protein expression found in neutrophils from COPD patients. Therefore, the peripheral neutrophil proteins regulated in COPD patients did not resemble TNFα- or GM-CSF-induced protein profiles. However, differential protein expression in neutrophils from COPD patients compared to age-matched healthy controls shows that using this technique a disease related neutrophil profile could be found.

## Methods

### Reagents

Ficoll-Paque was obtained from GE Healthcare (Uppsala, Sweden). Human serum albumin (HSA) was from Sanquin (Amsterdam, the Netherlands). Recombinant human TNFα was purchased from Roche (Indianapolis, IN). Recombinant human GM-CSF was a gift from Prof. A. Lopez (Institute of Medical and Veterinary Sciences, Adelaide, Australia). All other materials were reagent grade.

### Patients and healthy control subjects

We included 13 patients with a diagnosis of COPD according to the Global Initiative for Chronic Obstructive Lung Disease (GOLD) [1] and 6 healthy age-matched control subjects (see for demographics table 1). All patients had stable COPD without an exacerbation in the last four weeks before entering the study. Patients with other inflammatory conditions, heart failure and treatment with oral glucocorticosteroids were excluded. Dyspnea was rated with the Medical Research Council (MRC) scores [23]. The medical ethics committee of the University Medical Center Utrecht (Utrecht, The Netherlands) approved the study, and all subjects provided written informed consent.

**Table 1:** Characteristics of study subjects

	Control	COPD	
Characteristics	(n = 6)	(n = 13)	Statistics
Age. Yr	60.3 (3.5)	65.5 (2.5)	n.s.
Gender			
Male	5	11	
Female	1	2	
FEV_1_			
L	3.33 (0.44)	1.40 (0.16)	0,006
% predicted	104.5 (8.4)	46.8 (4.9)	< 0.001
FEV_1_/FVC ratio	78.0 (2.0)	45.4 (4.4)	< 0.001
GOLD			
II		5	
III		6	
IV		2	
MRC score			
0	6		
1		4	
2		4	
3		5	
Smoking status			
Current smokers		2	
Ex-smokers	3	11	
Never smokers	3		
Weight. kg	74.7 (3.6)	81.7 (3.8)	n.s.
Height. cm	175 (4)	178 (3)	n.s.
BMI. kg/m^2^	24.4 (0.8)	25.8 (0.9)	n.s.
hsCRP, mg/L	3.8 (2.4)	4.6 (1.8)	n.s.
Leukocyte cell count. x10^6 ^cells/mL	6.7 (0.8)	8.1 (0.5)	n.s.
Data is represented as mean ± standard error of the mean			

### Granulocyte isolation

Granulocytes were isolated from whole blood anticoagulated with sodium-heparin from COPD patients or age-matched healthy control subjects. Blood was diluted 2.5:1 with PBS containing trisodium citrate (0.4% w/v, pH 7.4) and human pasteurized plasma-protein solution (4 g/L). Mononuclear cells and granulocytes were separated by centrifugation using Ficoll-Paque. Erythrocytes were lysed in isotonic ice-cold NH_4_Cl solution (8.3 g/L NH_4_Cl, 1 g/L KHCO_3 _and 37 mg/L EDTA) followed by centrifugation at 4°C. After isolation, granulocytes were washed in PBS containing trisodium citrate (0.4% w/v, pH 7.4) and human pasteurized plasma-protein solution (4 g/L) and resuspended in HEPES buffered RPMI 1640 supplemented with 0.5% (w/v) HSA. Purity of neutrophils was >95% with eosinophils as major contaminant.

### Neutrophil stimulation and protein extracts preparation

Neutrophils (5 × 10^6^/mL) in HEPES buffered RPMI 1640 supplemented with 0.5% (w/v) HSA were incubated for 30 min at 37°C. Subsequently, neutrophils of COPD patients and healthy age-matched controls were immediately prepared for protein extracts (see below). Furthermore, neutrophils of healthy age-matched controls were incubated without cytokines or stimulated with TNFα (100 U/mL), GM-CSF (100 pM) or both for 4 hours at 37°C. All neutrophil samples (1.10^7^/sample) were washed twice (0.34 M sucrose, 1 mM EDTA, 10 mM Tris) and lysed in lysis buffer (10 mM Tris pH 7.4, 10% glycerol, 1% NP40, 50 mM NaF, 20 mM tetra-Na pyrophosphate, 1 mM DTT, 2 mM vanadate, 1 mM PMSF, 2 mM DFP and 1 × Complete EDTA-free protease inhibitor cocktail tablet (Roche)). Proteins were precipitated with 80% acetone and dissolved in labeling buffer (8 M Urea, 2 M Thiourea, 4% CHAPS, 10 mM Tris pH 8.5).

### CyDye labeling

The DIGE technology is based on differential protein labeling with different fluorescent CyDyes, which allows sample multiplexing. This method is an unbiased approach to identify differences in protein expression and the use of an internal standard enables identification of protein differences as small as 10% [24]. Protein extracts were labeled using the fluorescent cyanine dyes developed for 2D-DIGE technology (GE Healthcare) following manufacturer's protocol with some minor modifications. Protein extracts (30 μg) were labeled with 300 pmol of fluorescent dye (Cy2, Cy3, or Cy5). Protein samples from COPD patients, healthy control or *in-vitro *stimulated neutrophils were randomly labeled with Cy3 or Cy5. And each dye was used a similar number of times in each group to exclude effects of preferential labeling. An internal standard, created by pooling 15 μg of each protein sample, was labeled with Cy2. Labeling was stopped by adding lysine and equal volume of 2 × IEF buffer (8 M Urea, 2 M Thiourea, 4% CHAPS, 300 mM DTT, 1.0% IPG buffer 3-10NL, 0.004% Broomphenolblue) to each sample.

### 2D-gel electrophoresis and analysis

Two protein samples (Cy3 and Cy5) were mixed with the Cy2-labeled internal control. Protein samples were passively rehydrated into 24 cm pH 3-10 NL strips (GE Healthcare, Uppsala, Sweden) for 10 hours followed by isoelectric focusing using a manifold-equipped IPGphor IEF unit (GE Healthcare) according to the manufacturer's protocol. The cysteine sulfhydryls were reduced with 1.0% DTT and carbamidomethylated with 2.5% Iodoacetamide in equilibration buffer (30% glycerol, 2% SDS, 6 M urea, 75 mM Tris, pH 8.8). Second dimensional SDS-PAGE was performed on hand-cast 12% SDS-PAGE gels using low fluorescence glass plates. Electrophoresis was carried out at 0.2 watts/gel for 2 hours followed by 1 watts/gel until completion using an Ettan DALT-12 unit (GE Healthcare). Gels were scanned using a Typhoon 9410 imager at 100 μm resolution (GE Healthcare). Scan settings were optimized for a maximal signal of 85.000 counts. Gel images were cropped using ImageQuantTL 2003 (GE Healthcare), spot detection was performed with DeCyder 7.0 DIA (Difference In-gel Analysis) software (GE Healthcare) and gel images were matched using DeCyder 7.0 BVA (Biological Variation Analysis) software (GE Healthcare). Statistical analysis was performed using 1-ANOVA DeCyder 7.0 BVA. For 2D-gel analysis *p *< 0.01 was considered statistically significant.

### Statistical analysis

Statistical analysis of 2D-DIGE spot intensity was performed using DeCyder 7.0 BVA or EDA software (GE Healthcare, Uppsala, Sweden). Statistical analysis of patient characteristics was performed using an independent sample *t *tests with statistical software package SPSS 16.0.

## Results

### Neutrophils from COPD patients show differentially regulated proteins compared to healthy controls

We first tested the hypothesis whether systemic inflammation in COPD would be reflected by differences in protein expression compared to neutrophil protein expression from healthy control subjects. Therefore, we analyzed the neutrophil proteome from COPD patients and healthy age-matched control subjects by 2D-DIGE. We compared peripheral neutrophil protein expression of 6 healthy age-matched control subjects with those of 13 COPD patients (for demographics see table 1). No significant differences were present in age, weight, length, BMI or leukocyte count, whereas FEV_1_, and FEV_1_/FVC ratio were significantly different between the two groups. CRP and leukocyte counts were measured as markers for systemic inflammation but no significant differences were found.

**Figure 1:**
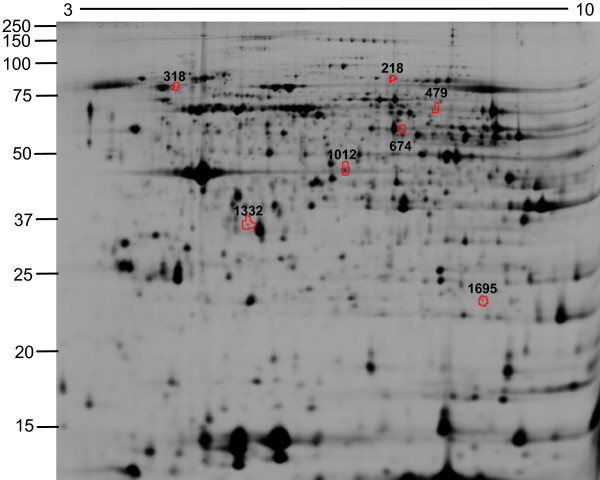
**2D-DIGE of peripheral neutrophils from COPD patients and healthy controls**. Neutrophils were isolated from the peripheral blood and protein lysates for 2D-DIGE were prepared. Proteins were focused on 24 cm pH 3-10 NL IEF strips and separated by 12% SDS-PAGE. Representative image of the biological variation analysis (BVA) software is shown. The differentially regulated proteins are indicated by the red spot boundaries.

**Figure 2:**
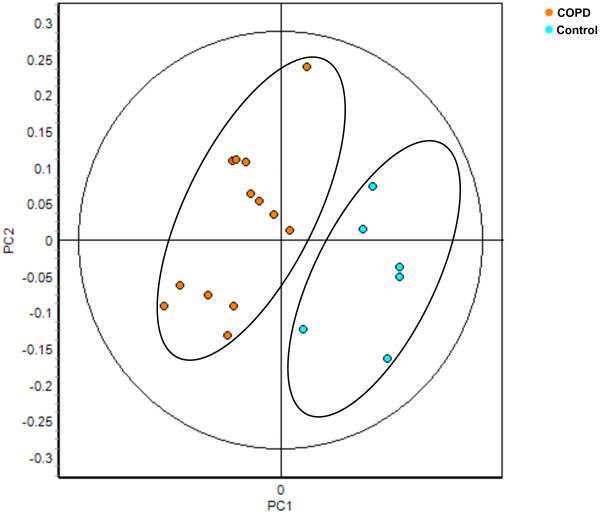
**COPD spotmaps were discriminative in a principal component analysis**. Data of the significant differential regulated proteins identified in the BVA of peripheral neutrophils from COPD patients (Spot ID 218, 318, 479, 674, 1012, 1332 and 1695) were imported into the extended data analysis (EDA) software. Principal component analysis (PCA) was performed on 6 healthy control (light blue) and 13 COPD (orange) spotmaps.

Next, we tested whether neutrophils from COPD patients showed significant protein differences compared to healthy controls. Neutrophil protein lysates of freshly isolated neutrophils from healthy controls or COPD patients were prepared, labeled with Cy3 or Cy5 and combined with an internal reference control stained with Cy2. Protein samples were separated by 2D-DIGE and analysis with DeCyder 7.0 identified 1200 - 2200 protein spots by a volume filter exclusion of 30.000 in the differential in-gel analysis (DIA) software. The individual spotmaps were matched in the biological variation analysis (BVA) software and statistical analysis between healthy controls and COPD patients showed 7 protein spots that were at least 1.10-fold differentially regulated with a *p *< 0.01 (Figure 1 and Table 2). The peripheral neutrophil spotmaps from COPD patients could be separated in a principal component analysis (PCA) from peripheral neutrophil spotmaps based on the differentially regulated proteins from healthy controls, showing that the differentially regulated proteins have a discriminatory power (Figure 2).

**Table 2:** Analysis of differentially regulated protein spots in neutrophils from COPD patients and *in-vitro* stimulated neutrophils

	No. regulated spots	
Control vs. COPD	7	
		
Not stimulated vs. TNFα	13	
Not stimulated vs. GM-CSF	20	
Not stimulated vs. TNFα + GM-CSF	22	
		

	No. regulated spots in COPD	No. regulated spots by GM-CSF

Control vs. COPD	7	0
Not stimulated vs. GM-CSF	0	20

### TNFα- and GM-CSF-modulated protein expression of human neutrophils

The differentially regulated proteins identified in neutrophils from COPD patients might have been induced upon exposure to dominant inflammatory mediators in the peripheral blood. We set out a study in which we compared the profile of neutrophils from COPD patients with *in vitro *cytokine-stimulated neutrophils to identify proteins that are similarly regulated and predict which cytokine(s) show(s) a predominant role in the systemic inflammation. We tested TNFα and GM-CSF because of their well-documented association with COPD [8,11,12,14-18]. Neutrophils from age-matched healthy controls were either left untreated or stimulated with TNFα (100 U/mL), GM-CSF (100 pM) or the combination for 4 hours at 37°C and, thereafter, protein lysates were made (n = 5). Subsequently, samples were labeled with Cy3 or Cy5 and were combined with an internal reference control stained with Cy2 and analyzed by 2D-DIGE. BVA analysis was performed as described above. Statistical analysis showed 13 protein spots to be differentially regulated more than 1.10-fold (1-ANOVA *p *< 0.01) following TNFα stimulation and 20 protein spots following GM-CSF stimulation, which included the 13 TNFα regulated protein spots (Table 2). Although TNFα did not show cytokine-specific regulated protein spots, it showed a potentiating effect on multiple GM-CSF-induced protein spots. The combination of TNFα and GM-CSF showed 22 differentially regulated protein spots. Two spots were specifically regulated more than 1.10-fold by the combination of TNFα + GM-CSF.

### Differentially regulated proteins in neutrophils from COPD patients do not correspond to differentially regulated protein spots in cytokine-stimulated neutrophils *in vitro*

**Figure 3:**
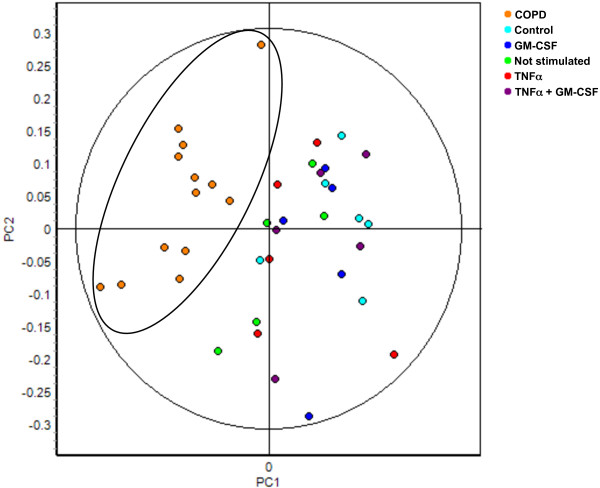
**COPD spotmaps did not show a GM-CSF or TNFα-induced protein profile in a principal component analysis**. Data of spot ID 218, 318, 479, 674, 1012, 1332 and 1695 (differentially regulated in COPD patients) from the COPD patient and *in vitro *stimulation BVA were imported into the EDA software. Principal component analysis (PCA) was performed on 5 non-stimulated (NS) (green), 5 TNFα-stimulated (red), 5 GM-CSF-stimulated (blue), 5 TNFα+GM-CSF-stimulated neutrophil spotmaps (purple), 6 healthy controls (light blue) and 13 COPD neutrophil spotmaps (orange).

GM-CSF and TNFα both induced expression of proteins in neutrophils *in vitro *and we tested the hypothesis whether these proteins corresponded to differentially regulated proteins in neutrophils from COPD patients. Seven proteins (Figure 1; Spot ID 218, 318, 479, 674, 1012, 1332 and 1695) were significantly different between neutrophils of healthy age-matched controls compared to neutrophils from COPD patients. These seven protein spots were traced back in the 2D-DIGE gels of *in vitro *stimulated neutrophils. Not any of these 7 protein spots showed differential regulation by GM-CSF (Table 2) or TNFα (data not shown). Vice versa, the 20 protein spots that were different between unstimulated neutrophils and GM-CSF stimulated neutrophils *in vitro *were not differentially regulated between neutrophils of healthy controls and COPD patients (Table 2). As a consequence, the COPD spotmaps (orange) did not cluster with TNFα-stimulated neutrophils (red) or GM-CSF-stimulated neutrophils (blue) in the PCA, based on the differentially regulated proteins in COPD patients (Figure 3). Therefore, this analysis shows that the protein profiles of COPD patients are not reflected by GM-CSF and/or TNFα-stimulated peripheral neutrophil profiles.

## Discussion

Increased neutrophil numbers and multiple inflammatory mediators have been found in sputum, bronchoalveolar lavage (BAL) fluid, bronchial biopsies and peripheral blood of COPD patients [8-12,25-27]. Therefore, we set out experiments to measure the neutrophil protein expression *ex vivo *as a means to identify cytokines that are dominant in the systemic inflammation in COPD patients. We have used 2D-DIGE, a novel technique that uses an internal reference sample in all 2D-gels, which enables the identification of protein expression differences as small as 10% [24]. Although the control group only includes 6 subjects, *in-vitro *stimulation of these neutrophils showed reproducible differences in protein expression. Furthermore, various 2D-DIGE publications have used similar group sizes [28-30]. Therefore, this technique is suitable to detect protein expression differences in relatively small study groups.

We performed for the first time 2D-DIGE on peripheral neutrophils from stable COPD patients and age-matched healthy control subjects and identified 7 significant (1-ANOVA *p *< 0.01) protein differences. A limitation of the study is the lack of an age-matched control group that smokes but do not have signs of COPD. The possible confounder in our study is that the differences found between healthy controls and COPD patients are smoking related and not disease related. Therefore, in additional PCA comparisons we excluded spotmaps from current smokers from the COPD group, which did not affect the clustering (data not shown). Also, comparing spotmaps from COPD patients with control spotmaps of either ex-smokers or non-smokers in the PCA showed clustering of COPD spotmaps apart from control spotmaps (data not shown). Based on these observations we find it unlikely that smoking is the reason for the differential neutrophil protein expression found in the COPD patients. Therefore, we hypothesize that the protein differences represent a baseline systemic inflammation in COPD patients. The protein differences found in neutrophils from COPD patients were subsequently compared with *in vitro *cytokine-stimulated neutrophils. For this approach, we selected *in vitro *stimulation with TNFα and/or GM-CSF because these cytokines are extensively described to be involved in the inflammation and pathogenesis of COPD. TNFα is an interesting cytokine in regard to systemic inflammation because it is linked to extrapulmonary manifestations of COPD such as osteopenia [31] and muscle wasting [32]. An important pathway induced by TNFα is the NF-κB pathway, and increased NF-κB activity has been shown in sputum neutrophils [33] and macrophages [34] of COPD patients. Also, GM-CSF is an important cytokine in the pathogenesis of COPD. Recently, Vlahos et. al. showed that mice exposed to cigarette smoke that were treated with neutralizing antibodies against GM-CSF exhibited reduced BAL fluid macrophages and neutrophils [13]. Also, TNFα, MIP-2 and MMP-12 mRNA levels were reduced in the lungs of anti-GM-CSF treated mice. This shows that GM-CSF is a key mediator in smoke-induced airway inflammation. We have previously shown that *in vitro *stimulation of neutrophils with either TNFα or GM-CSF resulted in differential expression of genes encoding for chemokines and cytokines [21]. In our current proteomics approach *in vitro *stimulation of peripheral neutrophils with TNFα or GM-CSF resulted in differential protein expression of 13 and 20 protein spots, respectively. All proteins regulated by TNFα were also regulated by GM-CSF, showing that both cytokines show redundancy in regulating protein expression. It is surprising that TNFα did not show cytokine-specific regulation of protein spots because it is an important stimulator for the NF-κB pathway, regulating multiple inflammatory mediators that are not regulated by GM-CSF [21]. Indeed, we previously showed that TNF-α increased the expression of chemokines in neutrophils on mRNA level *in vitro *[21]. Also, we have confirmed TNF-α-induced production of IL-1β on protein level in neutrophils *in vitro *[35]. A possible explanation could be that the expression levels of these inflammatory mediators in the neutrophils are too low to be detected by 2D-DIGE.

GM-CSF-induced protein expression in neutrophils *in vitro *was compared with protein expression of neutrophils from COPD patients. Protein differences that were found in neutrophils from COPD patients did not correspond to protein differences found in GM-CSF-stimulated neutrophils (Figure 3, Table 2). It is very well possible that other inflammatory mediators next to GM-CSF modulated protein expression in peripheral neutrophils from COPD patients. For instance, increased levels of IL-6, IL-8, and CRP were found in the peripheral blood op COPD patients [36,37]. Which of these factors, or combination of factors, play a role in the regulation of neutrophil proteins in COPD patients is currently not known. At least, we can exclude a prominent role for TNFα or GM-CSF on peripheral blood neutrophils. The identification of the differentially regulated neutrophil protein spots from COPD patients by mass spectrometry might delineate the inflammatory factors involved in neutrophil protein regulation *in vivo*, however, this was not the aim of this investigation.

Our data do not support the hypothesis that TNFα and GM-CSF drive systemic inflammation in stable COPD patients with a normal BMI (Table 1). Interestingly, we did not find increased hsCRP levels in our cohort of COPD patients, which is a marker for systemic inflammation. The most likely explanation for the low C-reactive protein (CRP) levels is the strict inclusion of stable COPD patients. Indeed, increased levels of acute phase proteins such as fibrinogen or CRP in COPD patients are mainly found during exacerbations [37-42]. Apparently, the differences found in the neutrophil proteome are not caused by an acute phase systemic inflammation since CRP levels were not significantly different between our included COPD patients and age-matched healthy control subjects.

## Conclusion

Systemic inflammation in COPD was determined by the analysis of the proteomes of peripheral blood neutrophils. The proteomes of the peripheral neutrophils of the COPD patients were not similar to peripheral neutrophils stimulated by TNFα and/or GM-CSF, neither did they correlate with increases in CRP. This indicates that systemic inflammation in COPD as visualized by peripheral neutrophil protein profiles is caused by a unique but subtle interplay between multiple inflammatory signals.

## Competing interests

The authors declare that they have no competing interests.

## Authors' contributions

JDL performed *in vitro *proteome preparation of neutrophils, analysis of neutrophil profiles using DeCyder software, statistical analysis and prepared the manuscript. RCS recruited and documented the subjects and assisted with the design of the study and manuscript preparation. JWJL assisted with the design of the study and manuscript preparation. LK conceived and designed the study, and assisted with manuscript preparation. LU designed the study and prepared manuscript and assisted with *in vitro *neutrophil preparations. All authors read and approved the manuscript.

## Pre-publication history

The pre-publication history for this paper can be accessed here:

http://www.biomedcentral.com/1471-2466/11/44/prepub
